# Supplementary material for: The Succession of the Cellulolytic Microbial Community from the Soil during Oat Straw Decomposition
Source: Int J Mol Sci. 2023 Mar 28;24(7):6342. doi: 10.3390/ijms24076342 (PMC10094526; doi:10.3390/ijms24076342)
Supplement: Supplementary file 1 [file ijms-24-06342-s001.zip › succession_figure_supplement_new2.pptx]

## Slide 1
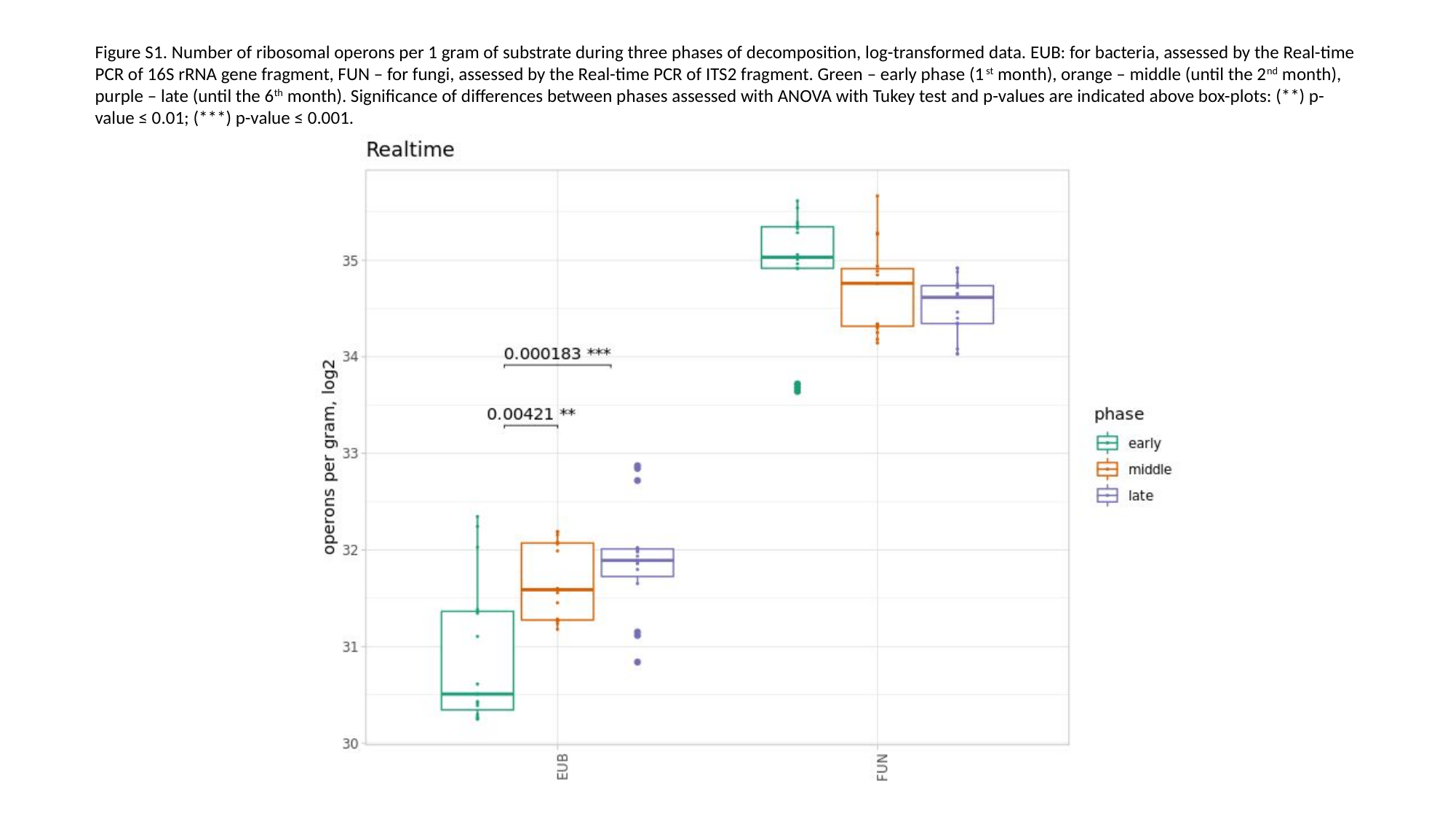

Figure S1. Number of ribosomal operons per 1 gram of substrate during three phases of decomposition, log-transformed data. EUB: for bacteria, assessed by the Real-time PCR of 16S rRNA gene fragment, FUN – for fungi, assessed by the Real-time PCR of ITS2 fragment. Green – early phase (1st month), orange – middle (until the 2nd month), purple – late (until the 6th month). Significance of differences between phases assessed with ANOVA with Tukey test and p-values are indicated above box-plots: (**) p-value ≤ 0.01; (***) p-value ≤ 0.001.

## Slide 2
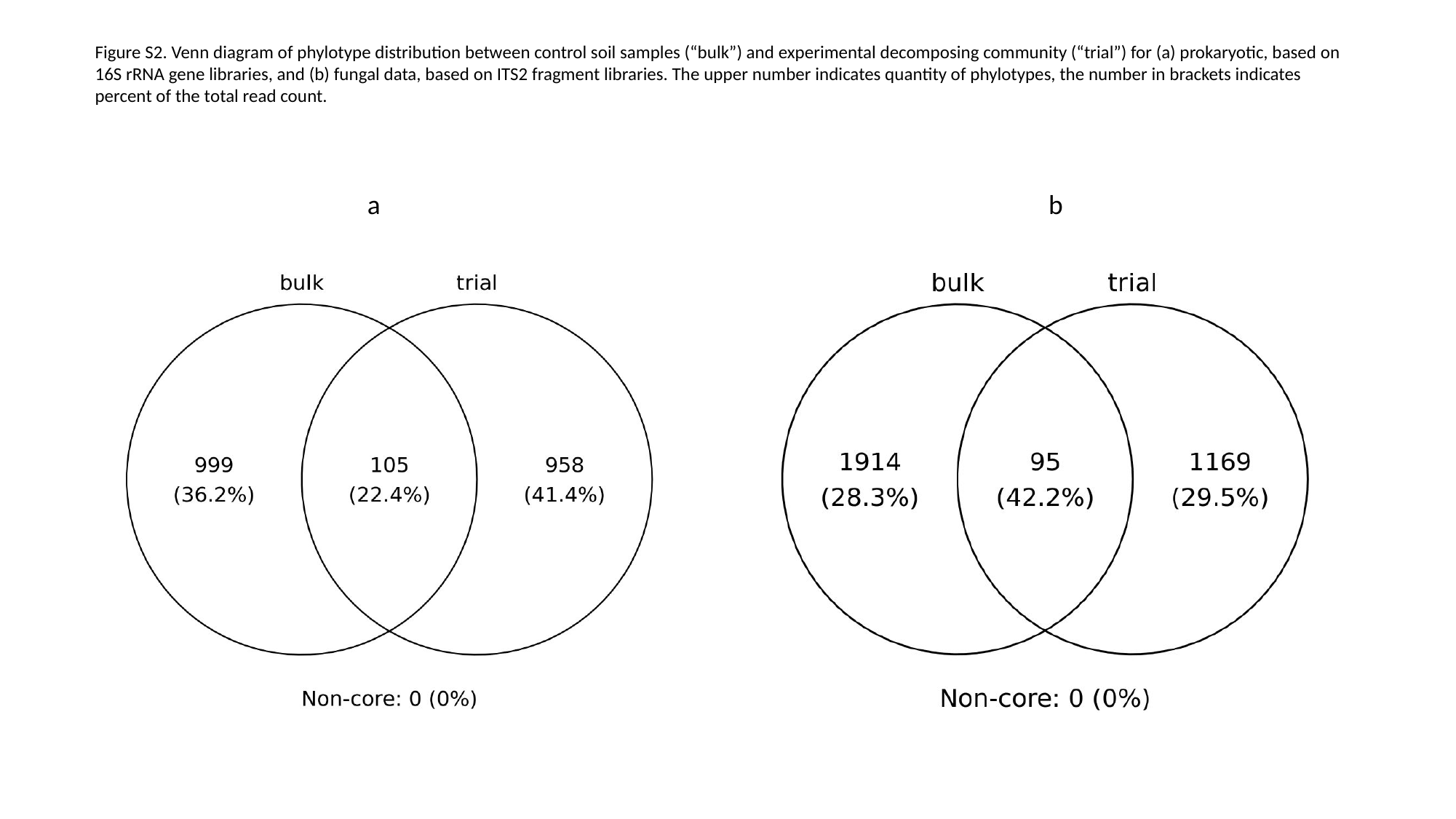

Figure S2. Venn diagram of phylotype distribution between control soil samples (“bulk”) and experimental decomposing community (“trial”) for (a) prokaryotic, based on 16S rRNA gene libraries, and (b) fungal data, based on ITS2 fragment libraries. The upper number indicates quantity of phylotypes, the number in brackets indicates percent of the total read count.
a b

## Slide 3
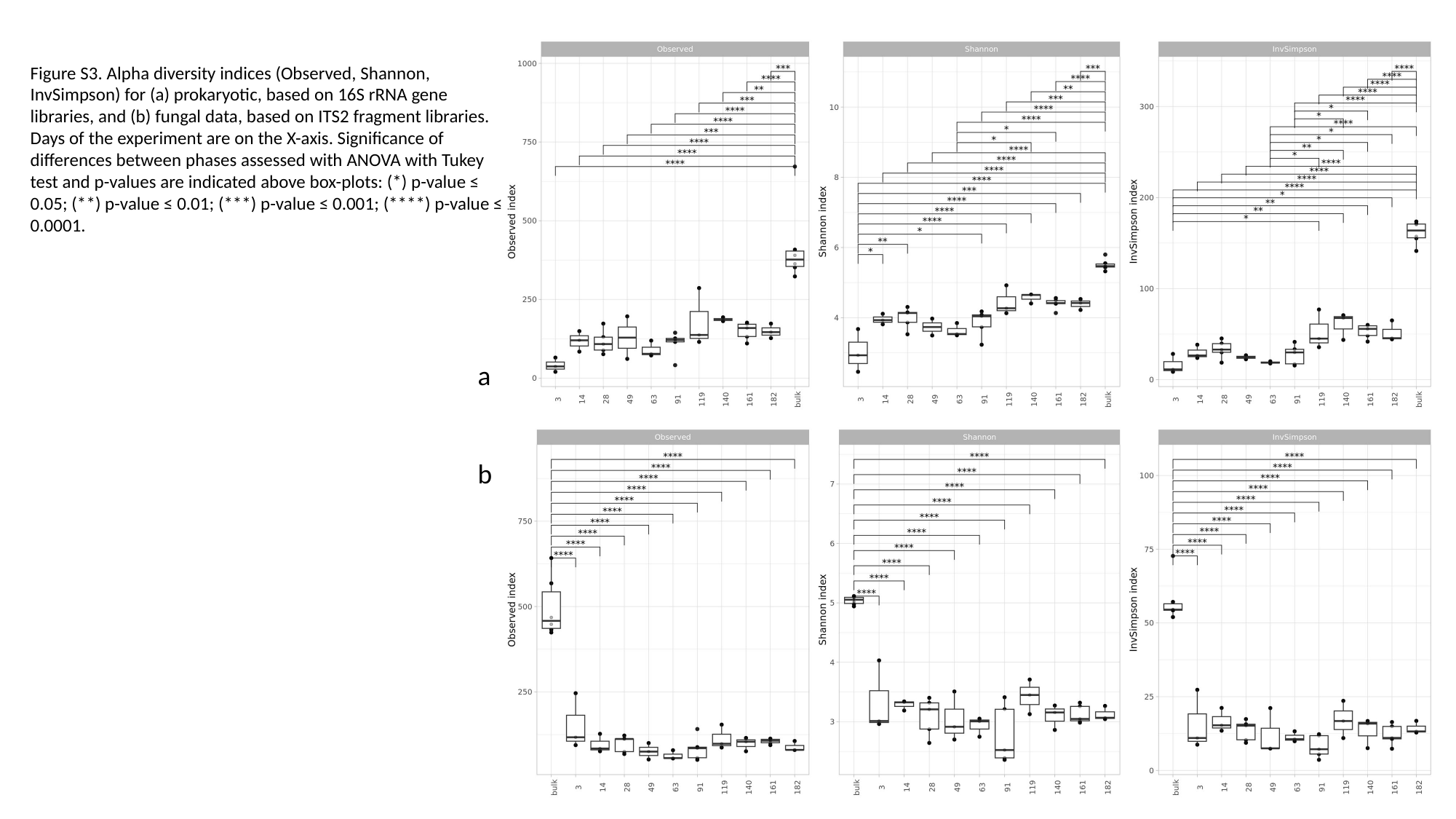

Figure S3. Alpha diversity indices (Observed, Shannon, InvSimpson) for (a) prokaryotic, based on 16S rRNA gene libraries, and (b) fungal data, based on ITS2 fragment libraries. Days of the experiment are on the X-axis. Significance of differences between phases assessed with ANOVA with Tukey test and p-values are indicated above box-plots: (*) p-value ≤ 0.05; (**) p-value ≤ 0.01; (***) p-value ≤ 0.001; (****) p-value ≤ 0.0001.
a
b

## Slide 4
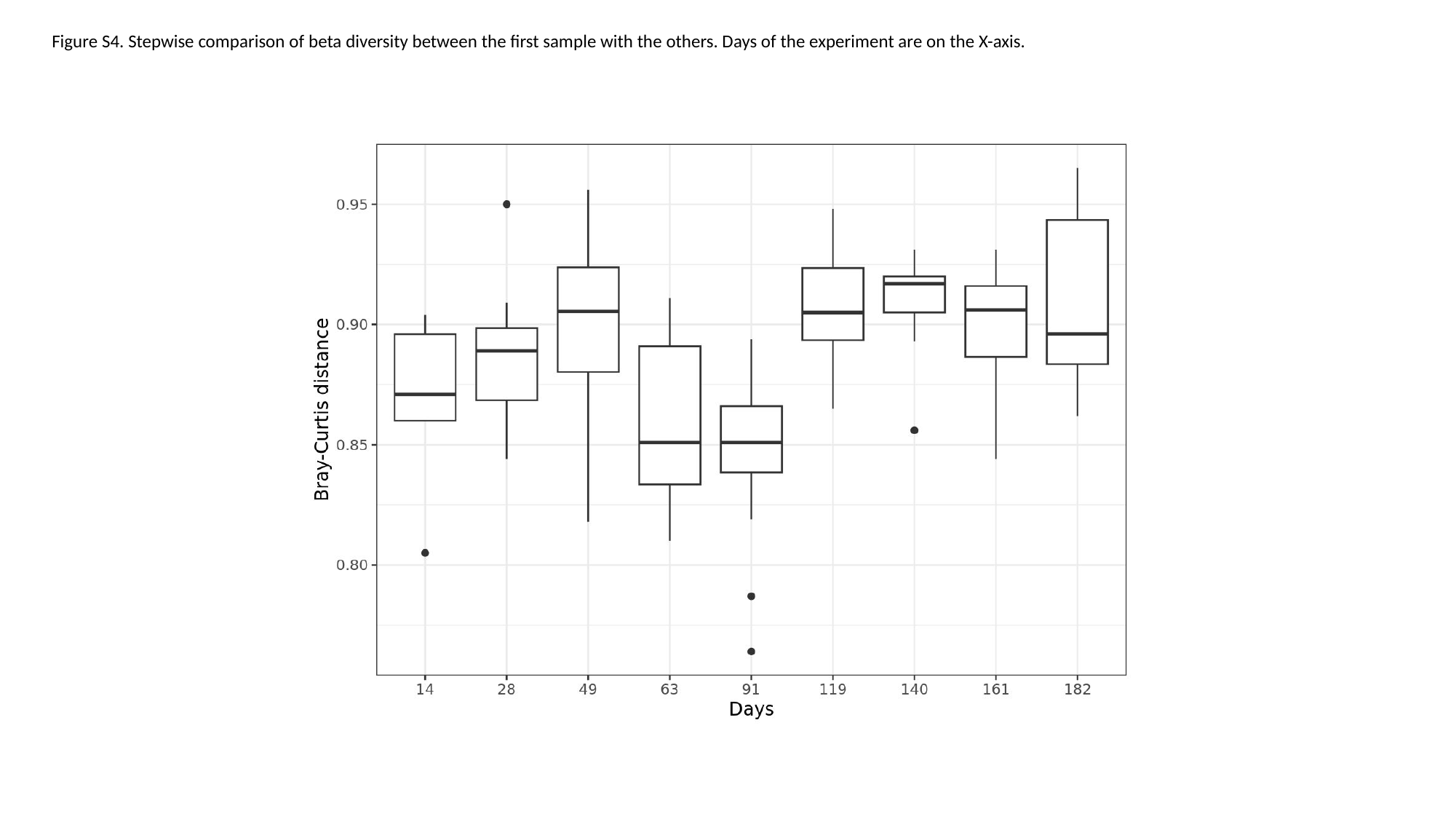

Figure S4. Stepwise comparison of beta diversity between the first sample with the others. Days of the experiment are on the X-axis.

## Slide 5
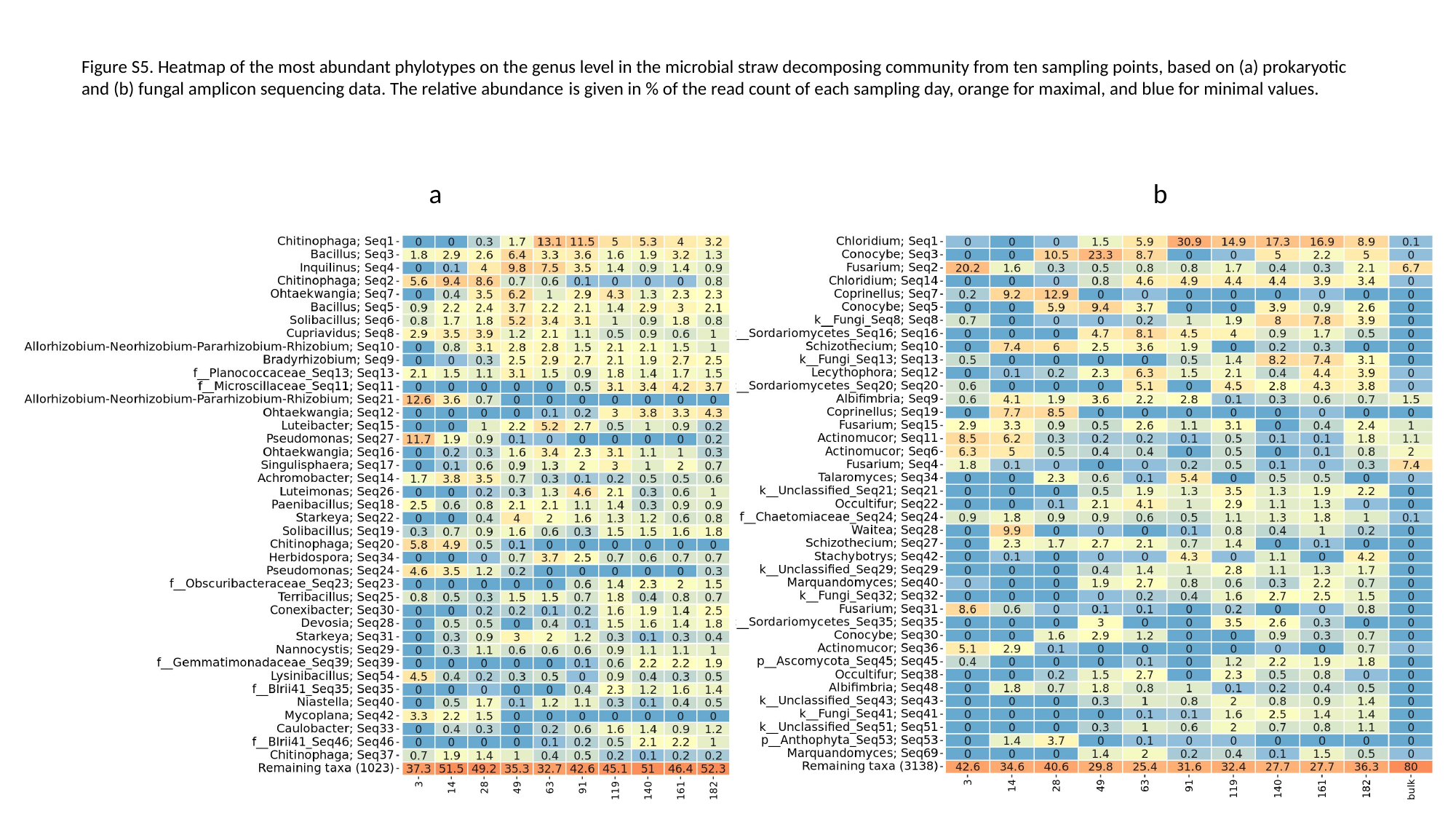

Figure S5. Heatmap of the most abundant phylotypes on the genus level in the microbial straw decomposing community from ten sampling points, based on (a) prokaryotic and (b) fungal amplicon sequencing data. The relative abundance is given in % of the read count of each sampling day, orange for maximal, and blue for minimal values.
a b

## Slide 6
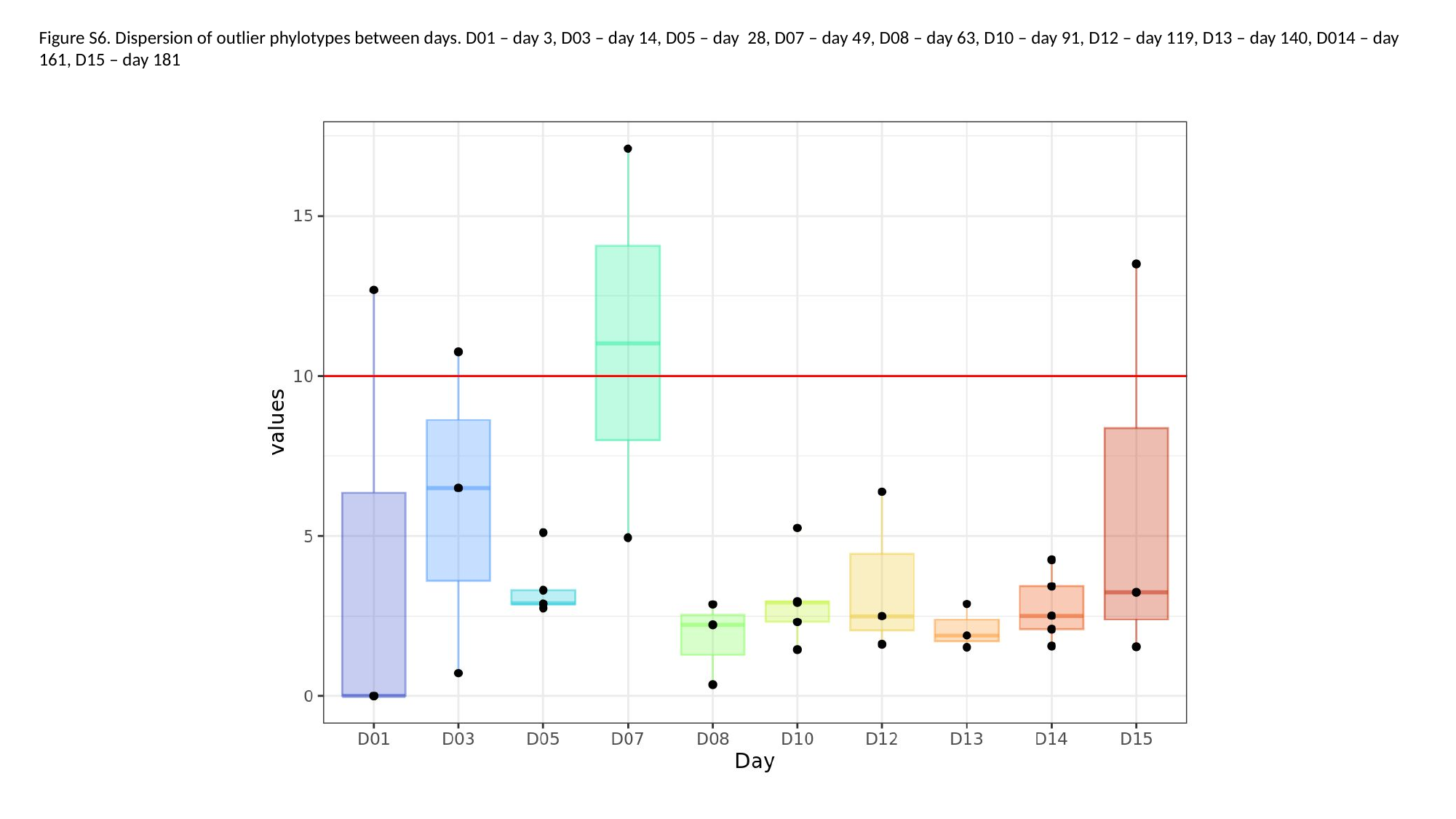

Figure S6. Dispersion of outlier phylotypes between days. D01 – day 3, D03 – day 14, D05 – day 28, D07 – day 49, D08 – day 63, D10 – day 91, D12 – day 119, D13 – day 140, D014 – day 161, D15 – day 181

## Slide 7
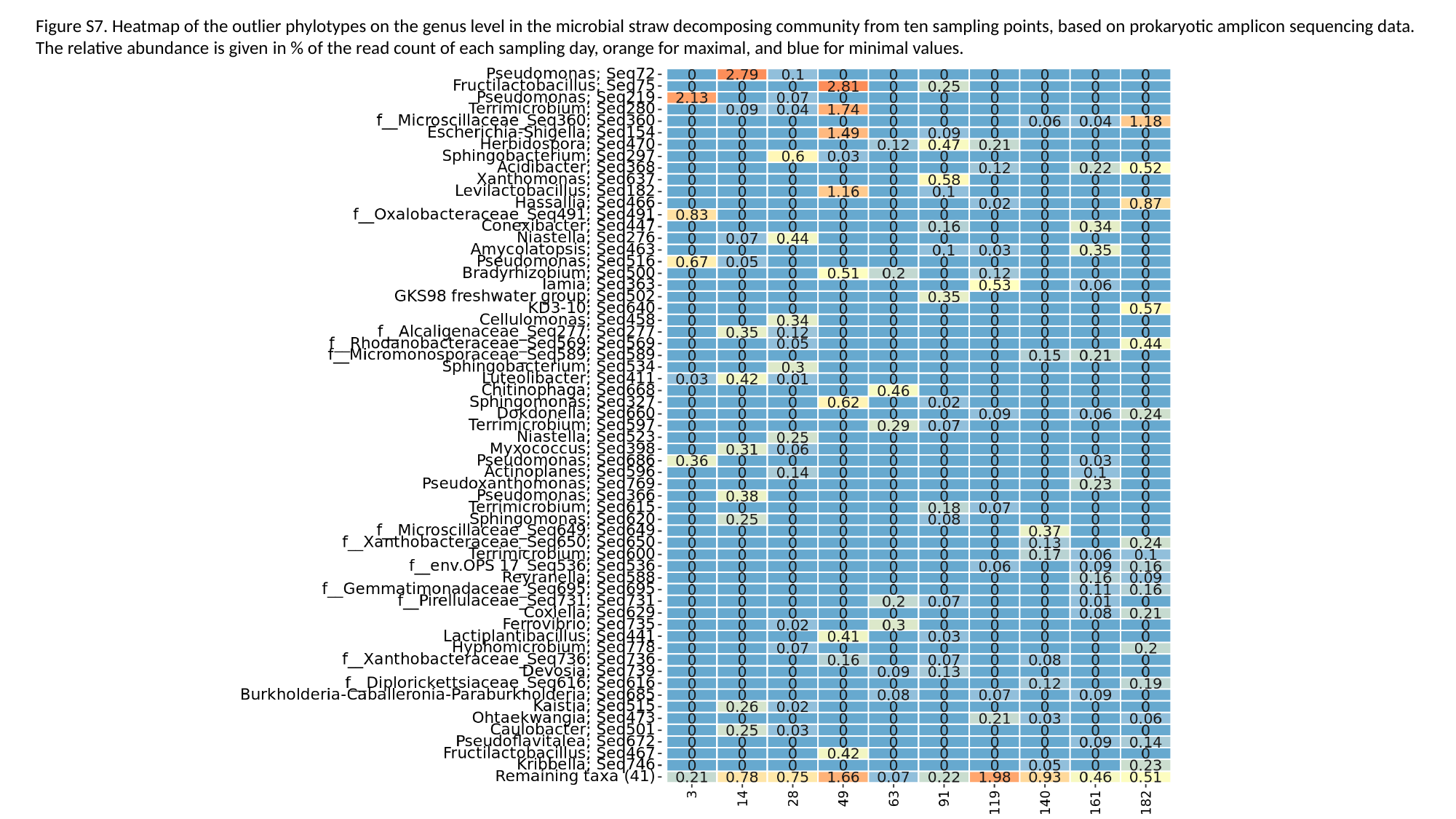

Figure S7. Heatmap of the outlier phylotypes on the genus level in the microbial straw decomposing community from ten sampling points, based on prokaryotic amplicon sequencing data. The relative abundance is given in % of the read count of each sampling day, orange for maximal, and blue for minimal values.

## Slide 8
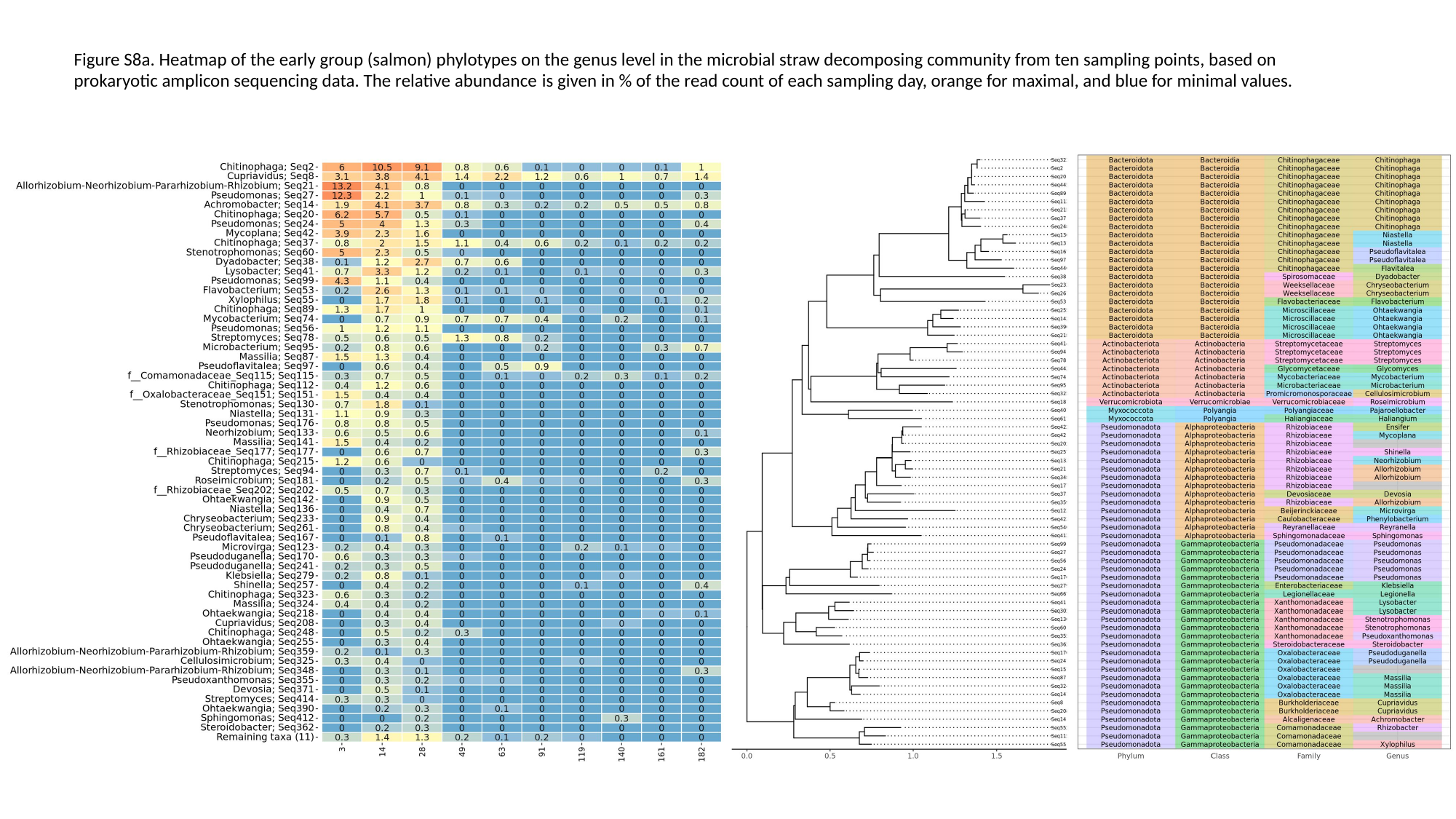

Figure S8a. Heatmap of the early group (salmon) phylotypes on the genus level in the microbial straw decomposing community from ten sampling points, based on prokaryotic amplicon sequencing data. The relative abundance is given in % of the read count of each sampling day, orange for maximal, and blue for minimal values.

## Slide 9
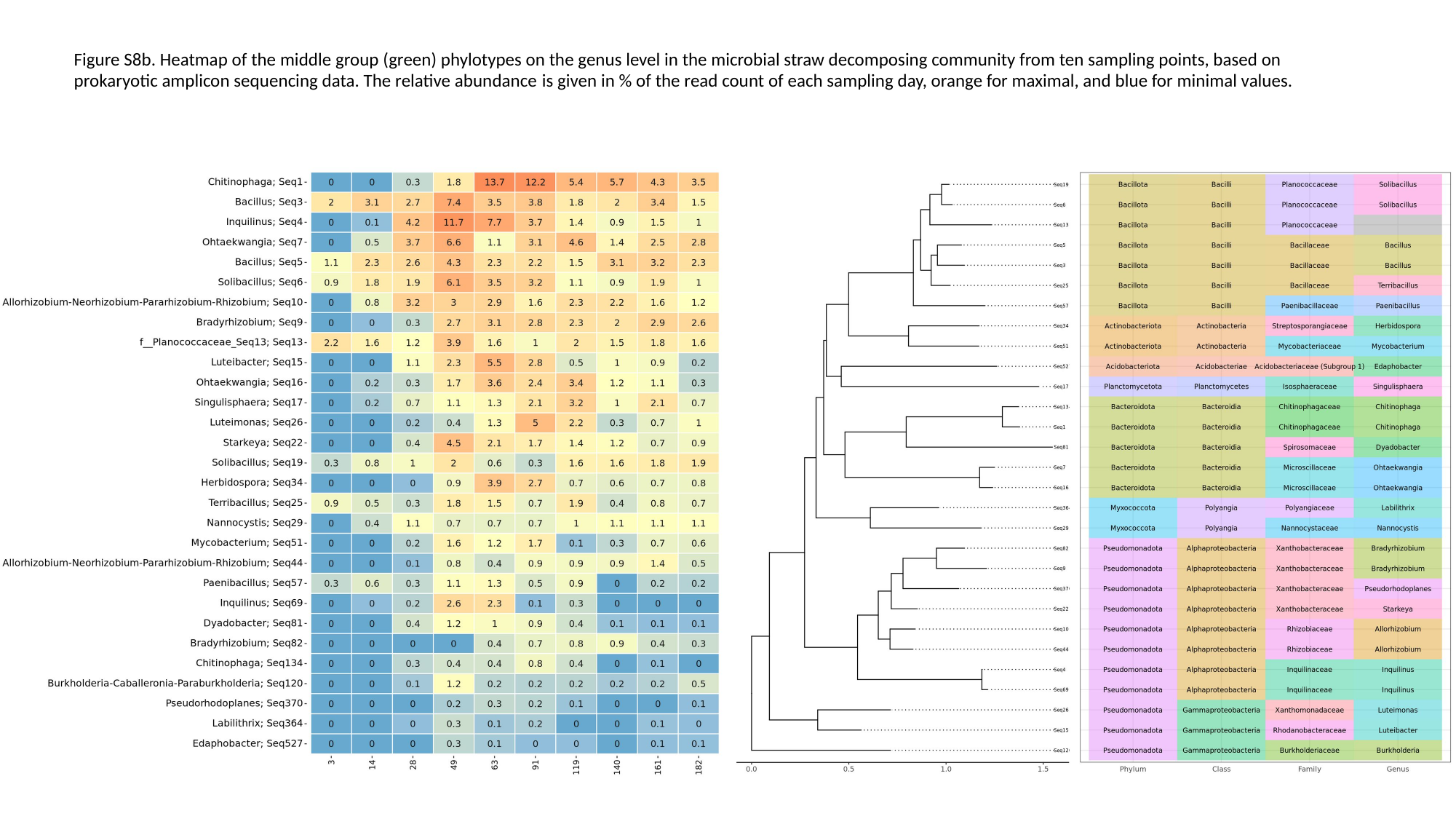

Figure S8b. Heatmap of the middle group (green) phylotypes on the genus level in the microbial straw decomposing community from ten sampling points, based on prokaryotic amplicon sequencing data. The relative abundance is given in % of the read count of each sampling day, orange for maximal, and blue for minimal values.

## Slide 10
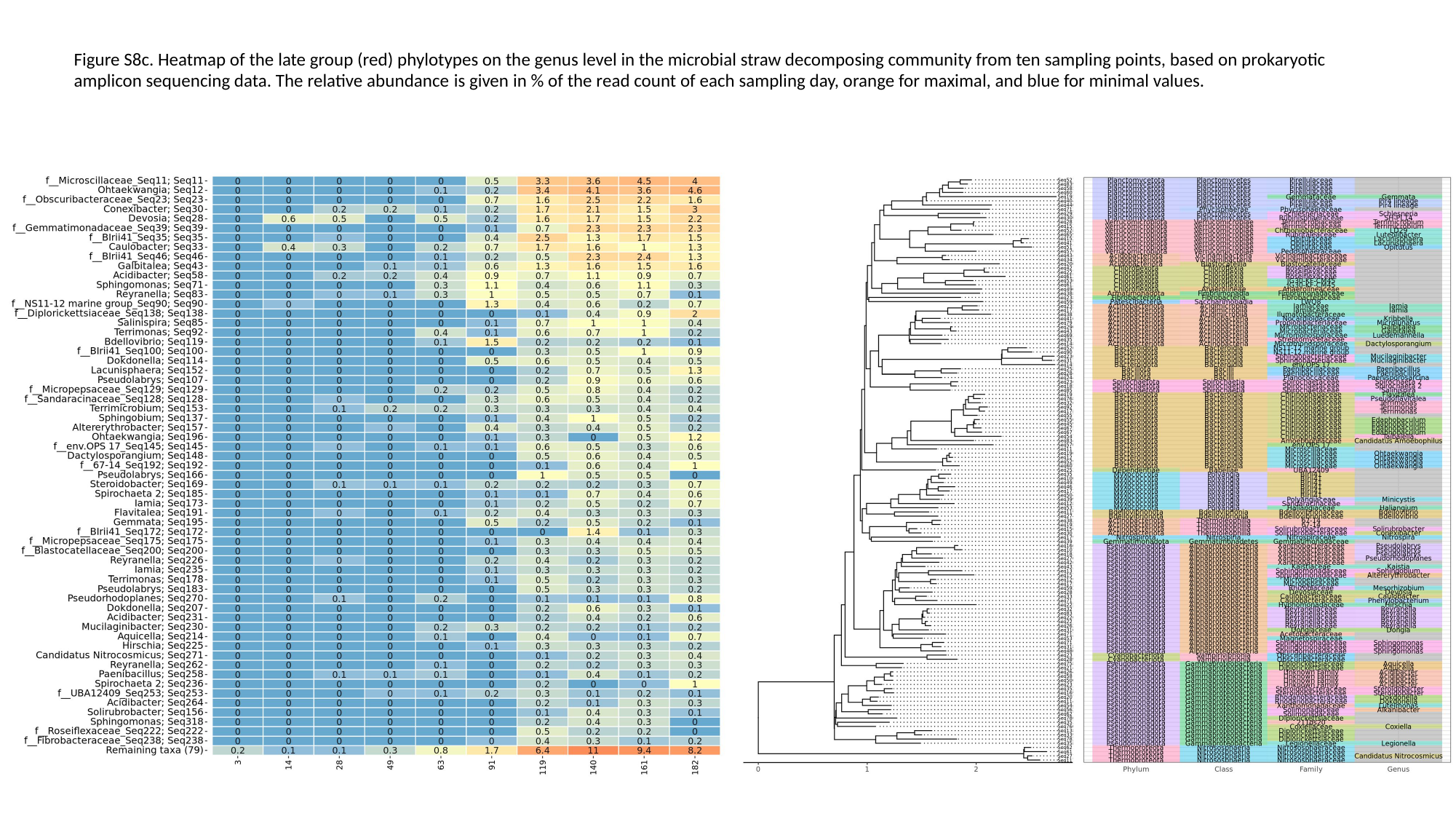

Figure S8c. Heatmap of the late group (red) phylotypes on the genus level in the microbial straw decomposing community from ten sampling points, based on prokaryotic amplicon sequencing data. The relative abundance is given in % of the read count of each sampling day, orange for maximal, and blue for minimal values.

## Slide 11
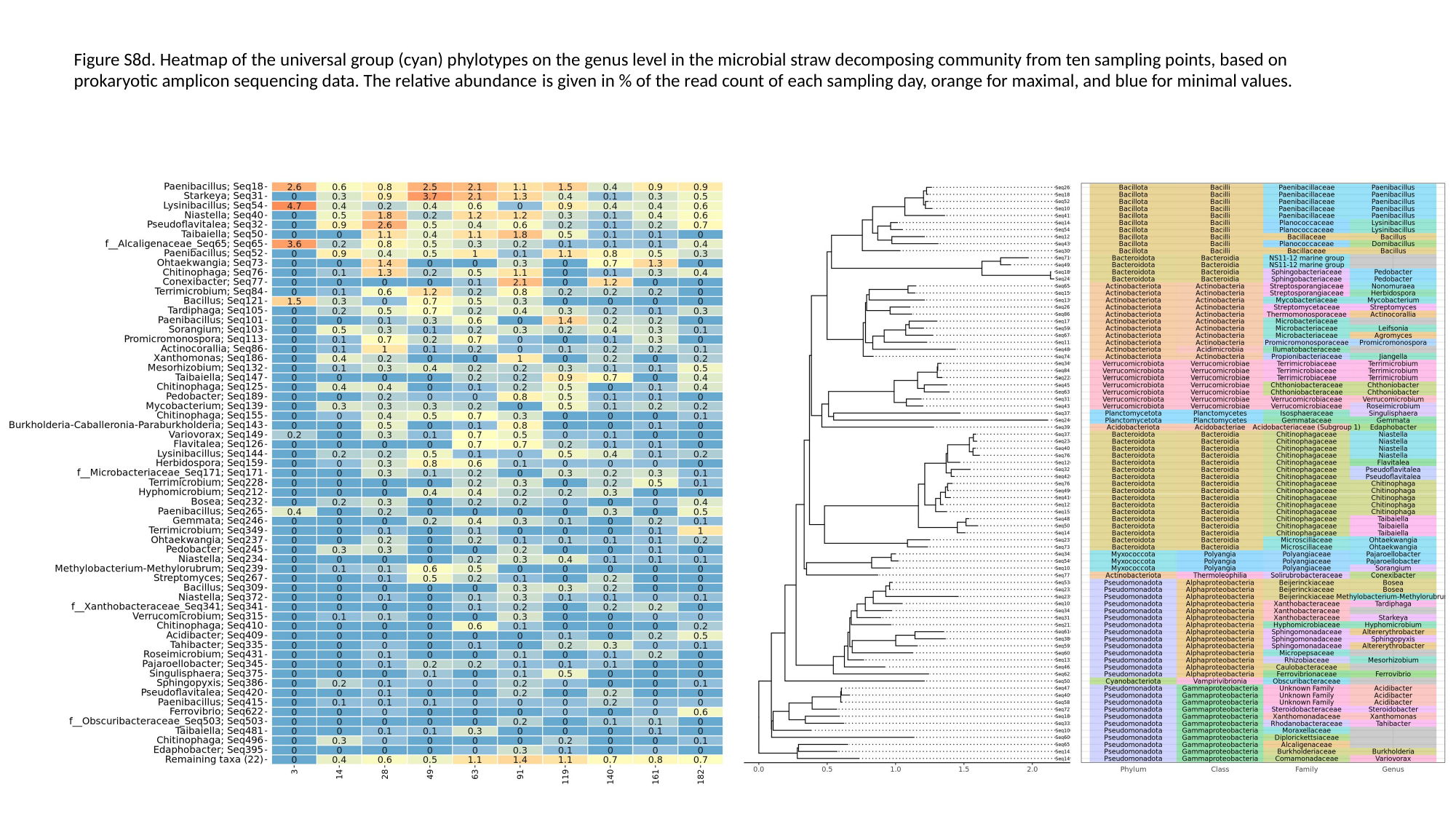

Figure S8d. Heatmap of the universal group (cyan) phylotypes on the genus level in the microbial straw decomposing community from ten sampling points, based on prokaryotic amplicon sequencing data. The relative abundance is given in % of the read count of each sampling day, orange for maximal, and blue for minimal values.

## Slide 12
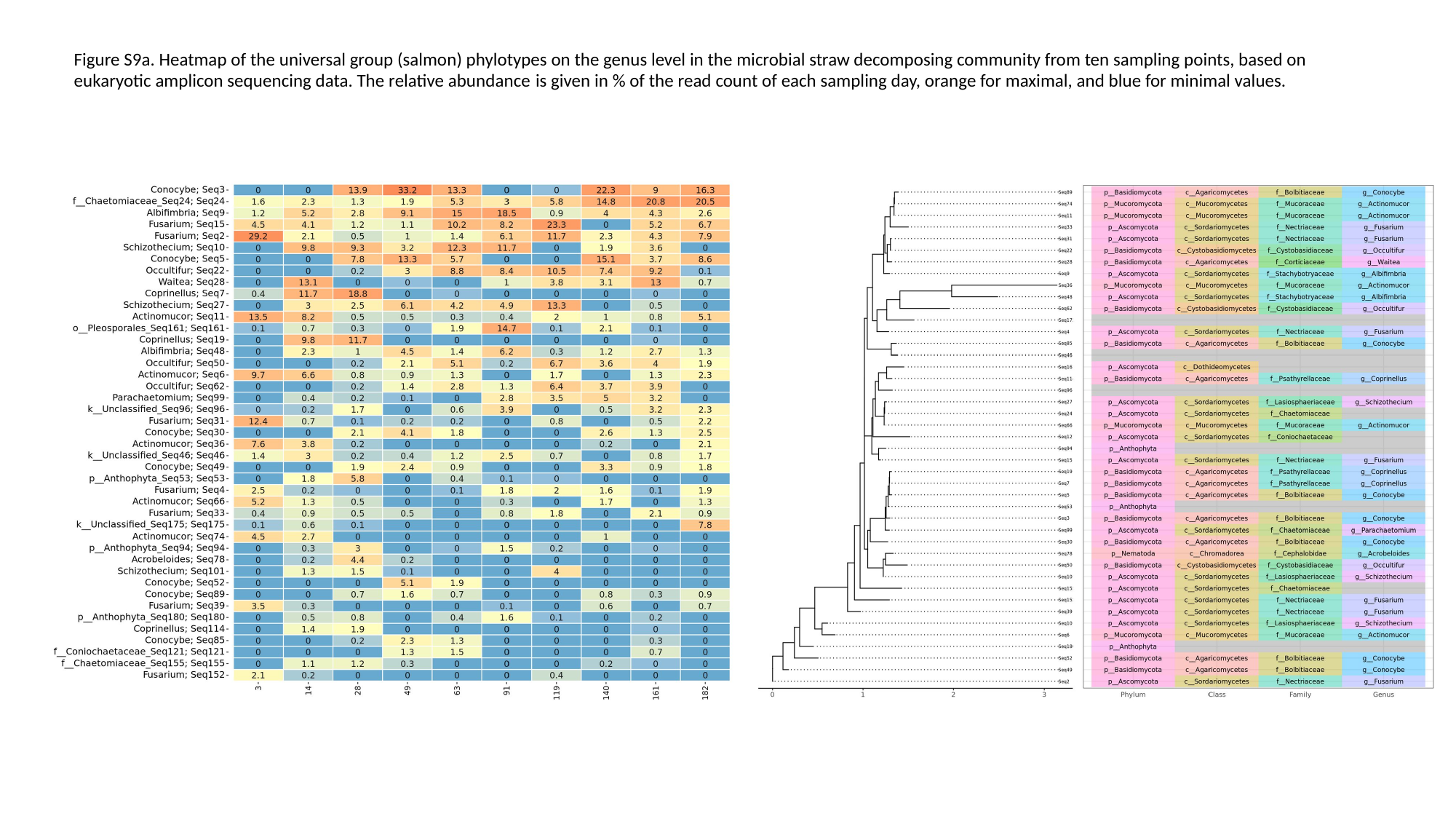

Figure S9a. Heatmap of the universal group (salmon) phylotypes on the genus level in the microbial straw decomposing community from ten sampling points, based on eukaryotic amplicon sequencing data. The relative abundance is given in % of the read count of each sampling day, orange for maximal, and blue for minimal values.

## Slide 13
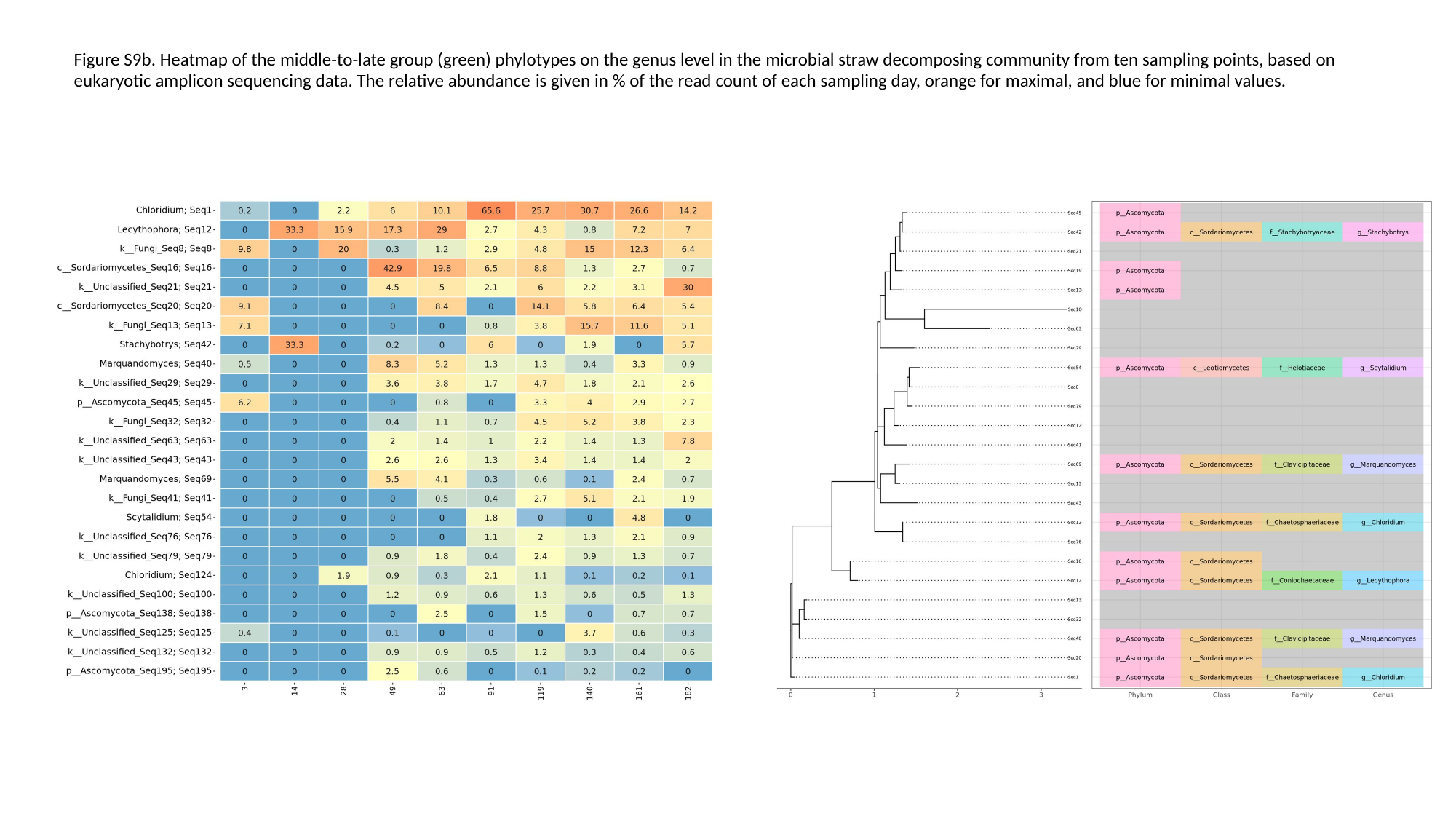

Figure S9b. Heatmap of the middle-to-late group (green) phylotypes on the genus level in the microbial straw decomposing community from ten sampling points, based on eukaryotic amplicon sequencing data. The relative abundance is given in % of the read count of each sampling day, orange for maximal, and blue for minimal values.

## Slide 14
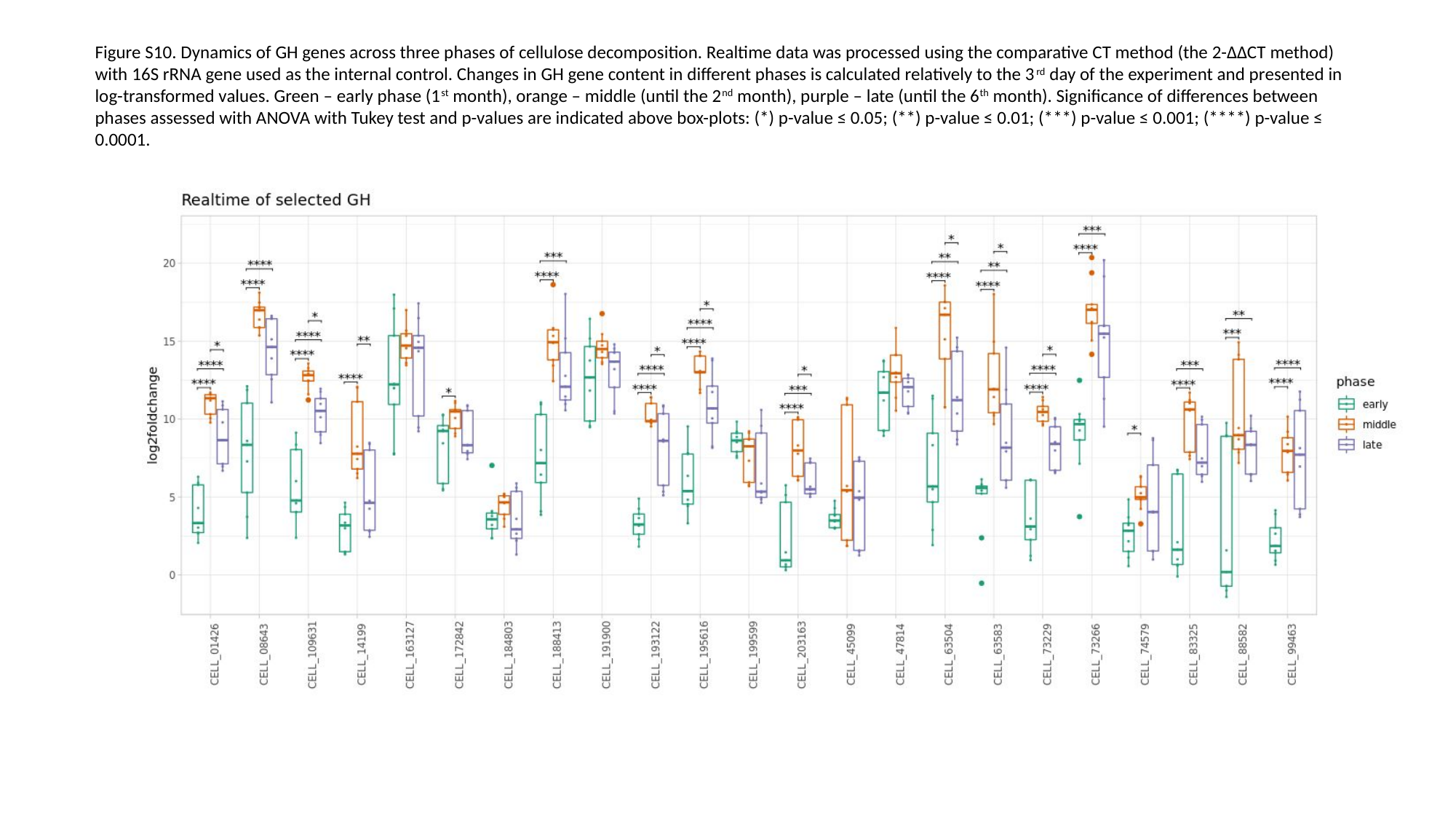

Figure S10. Dynamics of GH genes across three phases of cellulose decomposition. Realtime data was processed using the comparative CT method (the 2-ΔΔCT method) with 16S rRNA gene used as the internal control. Changes in GH gene content in different phases is calculated relatively to the 3rd day of the experiment and presented in log-transformed values. Green – early phase (1st month), orange – middle (until the 2nd month), purple – late (until the 6th month). Significance of differences between phases assessed with ANOVA with Tukey test and p-values are indicated above box-plots: (*) p-value ≤ 0.05; (**) p-value ≤ 0.01; (***) p-value ≤ 0.001; (****) p-value ≤ 0.0001.
